# T‐lymphocyte subtyping: an early warning and a potential prognostic indicator of active cytomegalovirus infection in patients with sepsis

**DOI:** 10.1111/imcb.12586

**Published:** 2022-10-12

**Authors:** Guangxu Bai, Na Cui, Hao Wang, Wei Cheng, Wen Han, Jianwei Chen, Ye Guo, Fei Wang

**Affiliations:** ^1^ Department of State Key Laboratory of Complex Severe and Rare Diseases, Peking Union Medical College Hospital Chinese Academy of Medical Science and Peking Union Medical College Beijing China; ^2^ Department of Critical Care Medicine, Peking Union Medical College Hospital, Peking Union Medical College Chinese Academy of Medical Sciences Beijing China; ^3^ Department of Clinical Laboratory, Peking Union Medical College Hospital, Peking Union Medical College Chinese Academy of Medical Science Beijing China; ^4^ Department of Critical Care Medicine Beijing Jishuitan Hospital Beijing China

**Keywords:** CMV‐DNA–negative conversion, cytomegalovirus, host immune phenotype, sepsis, T‐lymphocyte subtyping

## Abstract

Cytomegalovirus (CMV) infection is very common in patients suffering from sepsis and may cause poor prognosis. To explore the relationship between immune status of patients with sepsis and CMV infection, we assessed T lymphocyte subtyping and other commonly used clinical parameters in patients with sepsis upon admission to the intensive care unit (ICU) and evaluated their potential impact on diagnosis and outcomes of active CMV infection. In our study, 82 of 599 patients with sepsis were diagnosed with active CMV infection. The 28‐day mortality was higher in active CMV–infected than nonactive CMV–infected patients (20.7% *versus* 9.9%); 51of 82 active CMV–infected patients with sepsis were assessed to have CMV‐DNA–negative conversion, while 31 were persistently positive for CMV DNA. Higher CD8^+^CD28^+^ T‐cell counts at presentation were associated with CMV‐DNA–negative conversion and lower 28‐day mortality. The CMV‐DNA–negative conversion and 28‐day mortality of active CMV–infected patients with sepsis could be predicted using cutoff values of 151 (74.5% sensitivity and 87.1% specificity) and 64.5 (52.9% sensitivity and 92.3% specificity) CD8^+^CD28^+^ T cells mL^−1^ at ICU admission, respectively. Higher CD8^+^CD28^+^ T‐cell count was significantly associated with active CMV infection, higher CMV‐DNA–negative conversion and lower 28‐day mortality, which may be a potential marker for early warning of active CMV infection and outcome prediction.

## Graphical abstract

Active cytomegalovirus (CMV) infection may worsen the poor prognosis in patients with sepsis. CD28^+^CD8^+^ T‐cell count was significantly associated with active CMV infection. Higher CD28^+^CD8^+^ T‐cell count may predict higher CMV‐DNA–negative conversion. Patients with higher CD28^+^CD8^+^ T‐cell count had lower 28‐day mortality.

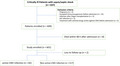

## INTRODUCTION

According to Sepsis 3.0,^1^ sepsis is defined as life‐threatening organ dysfunction caused by a dysregulated host response to infection. Host immune imbalance is the core mechanism for lethal organ dysfunction in patients with sepsis, and the main reason for susceptibility to multiple pathogens.^2^, ^3^ Cytomegalovirus (CMV) is the most common opportunistic virus in immunocompromised hosts, such as those with organ transplantation, HIV infection and long‐term chemotherapy,^4^, ^5^ and CMV infection is also common in patients suffering from sepsis.^6^ Studies have shown that the incidence of CMV infection in patients with sepsis during intensive care unit (ICU) hospitalization is as high as 15–30%.^7^ Compared with patients without CMV infection, the length of ICU stay and use of mechanical ventilation in patients with CMV infection remain significantly prolonged, and overall mortality doubles.^8^

The occurrence of CMV infection is associated with the level of host immunity.^9^ Studies have shown that the occurrence of CMV infection in patients with HIV infection or organ transplantation is closely associated with a significant decrease in natural killer (NK) and CD4^+^ T‐cell levels.^10^, ^11^ Thus, is there also a correlation between host immune imbalance and CMV infection in patients with sepsis? Does this immune imbalance affect the clinical diagnosis and treatment of CMV infection and even the prognosis? Currently, our knowledge of these issues remains limited. Therefore, in this study, we collected data related to CMV infection in patients with sepsis. We explored the correlation between host immune phenotype and clinical diagnosis and prognosis of sepsis, by assessing with immune indicators such as T cells, immunoglobulin and complement.

## RESULTS

### Characteristics of patients

**Figure 1:**
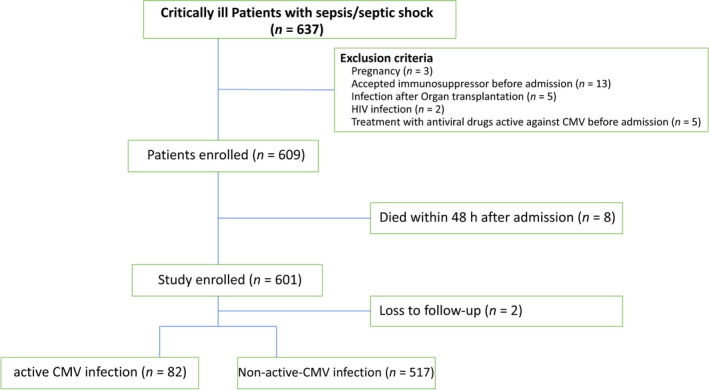
Flowchart of patient enrollment. CMV, cytomegalovirus.

During the study, a total of 637 patients with sepsis were admitted to our ICU. Of these, 28 were excluded from this study analysis according to the exclusion criteria, 8 died within 48 h, 3 were pregnant and 2 were lost to follow‐up. The remaining 599 patients with sepsis were enrolled in the study, of which 82 patients had active CMV infection and 517 had no active CMV infection (Figure 1). As CMV may remain in a latent state in humans, we used “nonactive CMV infection,” which includes both latent CMV and CMV negative, to group the enrolled patients. Tables 1 and 2 show the baseline and clinical characteristics of enrolled patients. No significant differences in sex, age, underlying diseases, Sequential Organ Failure Assessment score, infection foci, serum biochemical parameters and infection markers were observed between the groups. Compared with nonactive CMV–infected patients with sepsis, the active CMV–infected group had higher Acute Physiology and Chronic Health Evaluation II scores, proportion of accompanying fungal infection, more use of antiviral drugs and higher Clinical Pulmonary Infection Score. The length of ICU stay, ICU mortality, in‐hospital mortality and 28‐day mortality in active CMV–infected patients with sepsis were also higher than those in the nonactive CMV–infected group. No significant difference in the length of hospital stay was found between the two groups.

**Table 1:** Baseline characteristics of the study population

Variables	Sepsis (*n* = 599)	CMV‐DNA+ (*n* = 82)	CMV‐DNA– (*n* = 517)	*P*‐value
Age (years), median (IQR)	63 (20)	66 (17)	63 (20)	0.204
Sex (male:female), *n*	376:223	55:27	321:196	0.386
Underlying disease, *n* (%)					
COPD	20 (3.3)	4 (4.9)	16 (3.1)	0.404
Heart failure	177 (29.5)	25 (30.5)	152 (29.4)	0.841
Diabetes mellitus	158 (26.4)	28 (34.1)	130 (25.1)	0.086
Liver cirrhosis	16 (2.7)	2 (2.4)	14 (2.7)	0.888
Tumor	117 (19.5)	10 (12.2)	107 (20.7)	0.071
Chronic renal failure	60 (10.0)	7 (8.5)	53 (10.3)	0.631
APACHE II score	17 (9)	18.5 (8)	16 (10)	**< 0.0001**
SOFA score	11 (6)	11 (5)	11 (6)	0.534

APACHE II, Acute Physiology and Chronic Health Evaluation II; CMV, cytomegalovirus; COPD, chronic obstructive pulmonary disease; IQR, interquartile range; SOFA, Sequential Organ Failure Assessment; bold values in Tables 1‐8 highlight the variables that were considered to be statistically significant.

Bold values are statistically significant.

**Table 2:** Clinical characteristics of the patients with sepsis

Variables	Sepsis (*n* = 599)	CMV‐DNA+ (*n* = 82)	CMV‐DNA– (*n* = 517)	*P*‐value
Life‐sustaining treatments, *n* (%)					
Mechanical ventilation	547 (91.3)	80 (97.6)	488 (94.4)	0.229
Need for vasopressor	541 (88.5)	73 (89.3)	457 (88.4)	0.868
Need for RRT	140 (23.4)	17 (20.7)	123 (23.8)	0.543
Sites of infection, *n* (%)					
Lung	274 (45.7)	32 (39.0)	242 (46.8)	0.189
Abdominal	80 (13.4)	7 (8.5)	73 (14.1)	0.167
Bloodstream	76 (12.7)	11 (13.4)	65 (12.5)	0.831
Skin and soft tissue	30 (5.0)	3 (3.7)	27 (5.2)	0.546
Others	9 (1.5)	1 (1.2)	8 (1.5)	0.821
Multiple site infection, *n* (%)					
Lung + Blood	53 (9.5)	11 (13.4)	42 (8.1)	0.117
Lung+ Abdominal	39 (6.5)	8 (9.8)	31 (6.0)	0.200
Others	38 (6.3)	9 (11.0)	29 (5.6)	0.064
Pathogens, *n* (%)					
Bacteria	495 (83)	68 (83.1)	427 (82.9)	0.941
Fungi	73 (12.2)	16 (19.5)	57 (11.0)	**0.029**
Others	37 (6.2)	6 (7.3)	31 (6.0)	0.644
Drug therapy, *n* (%)					
Antifungal drugs	112 (18.7)	21 (25.6)	91 (17.6)	0.084	
Antiviral drugs	107 (17.9)	82 (100)	25 (4.8)	**< 0.0001**	
Biochemical parameters at ICU admission, median (IQR)					
Albumin (g L^−1^)	32 (6)	32 (7)	32 (6)	0.557	
Serum creatinine (μmol L^−1^)	86 (90)	95.5 (80)	85 (93)	0.058	
Total bilirubin (μmol L^−1^)	17.6 (13.2)	19.9 (39)	17.4 (11.2)	0.156	
Infection marker at ICU admission, median (IQR)					
Procalcitonin (ng mL^−1^)	2.3 (7.0)	1.79 (5.11)	2.45 (7.32)	0.12	
CPIS	6 (4)	6.5 (2)	6 (4)	**0.036**	
Outcome					
ICU durations (days), median (IQR)	10 (13)	16.5 (22)	9 (11)	< 0.0001	
Hospital durations (days), median (IQR)	20 (23)	23 (23)	19 (23)	0.114	
ICU mortality, *n* (%)	71 (11.9)	17 (20.7)	54 (10.4)	**0.007**	
Hospital mortality, *n* (%)	84 (14)	19 (23.2)	65 (12.6)	**0.010**	
28 days mortality, *n* (%)	68 (11.4)	17 (20.7)	51 (9.9)	**0.004**	

CMV, cytomegalovirus; CPIS, Clinical Pulmonary Infection Score; ICU, intensive care unit; IQR, interquartile range; RRT, Renal Replacement Therapy.

Bold values are statistically significant.

### Comparison of immune parameters in active CMV– and nonactive CMV–infected patients

Table 3 shows the immune parameters of all the patients at the time of enrollment. No significant differences in most immune parameters were observed between the two groups. However, NK cell count, CD8^+^ T‐cell count, CD28^+^ T‐cell count and immunoglobulin G level in active CMV–infected patients with sepsis had significant differences compared with those in the nonactive CMV–infected group. As the CD8^+^ T‐cell count is the sum of the CD8^+^CD28^+^ and CD28^−^CD8^+^ T‐cell count, these variables were considered collinear, and so we used CD8^+^ and CD8^+^CD28^+^ T‐cell counts in the multivariate logistic regression with other significantly different variables. The regression analysis showed that both the CD8^+^CD28^+^ and CD8^+^ T‐cell counts could be combined with the NK cell count, Acute Physiology and Chronic Health Evaluation II score and immunoglobulin G as related factors for active CMV infection in patients with sepsis (Table 4 and Supplementary table 1).

**Table 3:** Immune parameters between patients with or without CMV infection

Variables	Sepsis (*n* = 599)	CMV‐DNA+ (*n* = 82)	CMV‐DNA– (*n* = 517)	*P‐*value
WBC (10^9^ L^−1^)	11.79 (8.9)	10.45 (8.31)	12.07 (9)	0.112
NG (10^9^ L^−1^)	9.95 (8.27)	8.37 (7.50)	10.04 (8.63)	0.154
NK (cells mm^−3^)	74 (85)	59.5 (54)	76 (90)	**0.002**
B cell (cells mm^−3^)	106 (119)	96 (87)	106 (123)	0.071
Lymphocyte (cells mm^−3^)	927 (616)	901 (532)	927 (629)	0.891
CD4^+^ T	363 (339)	399 (352)	358 (338)	0.471
CD4^+^CD28^+^ T	337 (342)	358.5 (384)	331 (333)	> 0.99
CD4^+^CD28^−^ T	10 (24)	10 (55)	10 (24)	0.282
CD8^+^T	278 (280)	361.5 (241)	266 (311)	**0.011**
CD8^+^CD28^+^T	99 (133)	148.5 (226)	96.5 (93)	**< 0.0001**
CD8^+^CD28^−^ T	172 (138)	158.5 (165)	153 (212)	0.847
Complement factor (g L^−1^)					
C3	0.78 (0.40)	0.79 (0.40)	0.78 (0.41)	0.632
C4	0.16 (0.10)	0.17 (0.1)	0.16 (0.10)	0.766
Immunoglobulin (g L^−1^)					
IgA	2.16 (1.29)	2.21 (1.76)	2.11 (1.30)	0.837
IgG	9.66 (5.76)	11.04 (8.64)	9.24 (5.31)	**0.026**
IgM	0.72 (0.52)	0.74 (0.56)	0.68 (0.53)	0.923

Data are presented as *n* (%) or median (IQR).

CMV, cytomegalovirus; Ig, immunoglobulin; IQR, interquartile range; NG, neutrophil granulocyte; NK, natural killer (cells); WBC, white blood cell.

Bold values are statistically significant.

**Table 4:** Multivariate logistic regression analysis of factors distinguishing CMV infection

Parameters	OR	95% CI	*P*‐value
Fungal infection	0.538	0.278–1.042	0.066
CPIS	1.093	0.992–1.204	0.071
APACHE II	1.046	1.009–1.085	**0.014**
NK	0.992	0.988–0.997	**0.001**
IgG	1.074	1.026–1.124	**0.002**
CD8^+^CD28^+^ T‐cell count	1.004	1.002–1.006	**< 0.0001**

APACHE II, Acute Physiology and Chronic Health Evaluation II; CI, confidence interval; CMV, cytomegalovirus; CPIS, Clinical Pulmonary Infection Score; IgG, immunoglobulin G; NK, natural killer (cells); OR, odds ratio.

Bold values are statistically significant.

### Comparison of immune parameters in CMV‐DNA persistently positive and negative conversion patients

**Figure 2:**
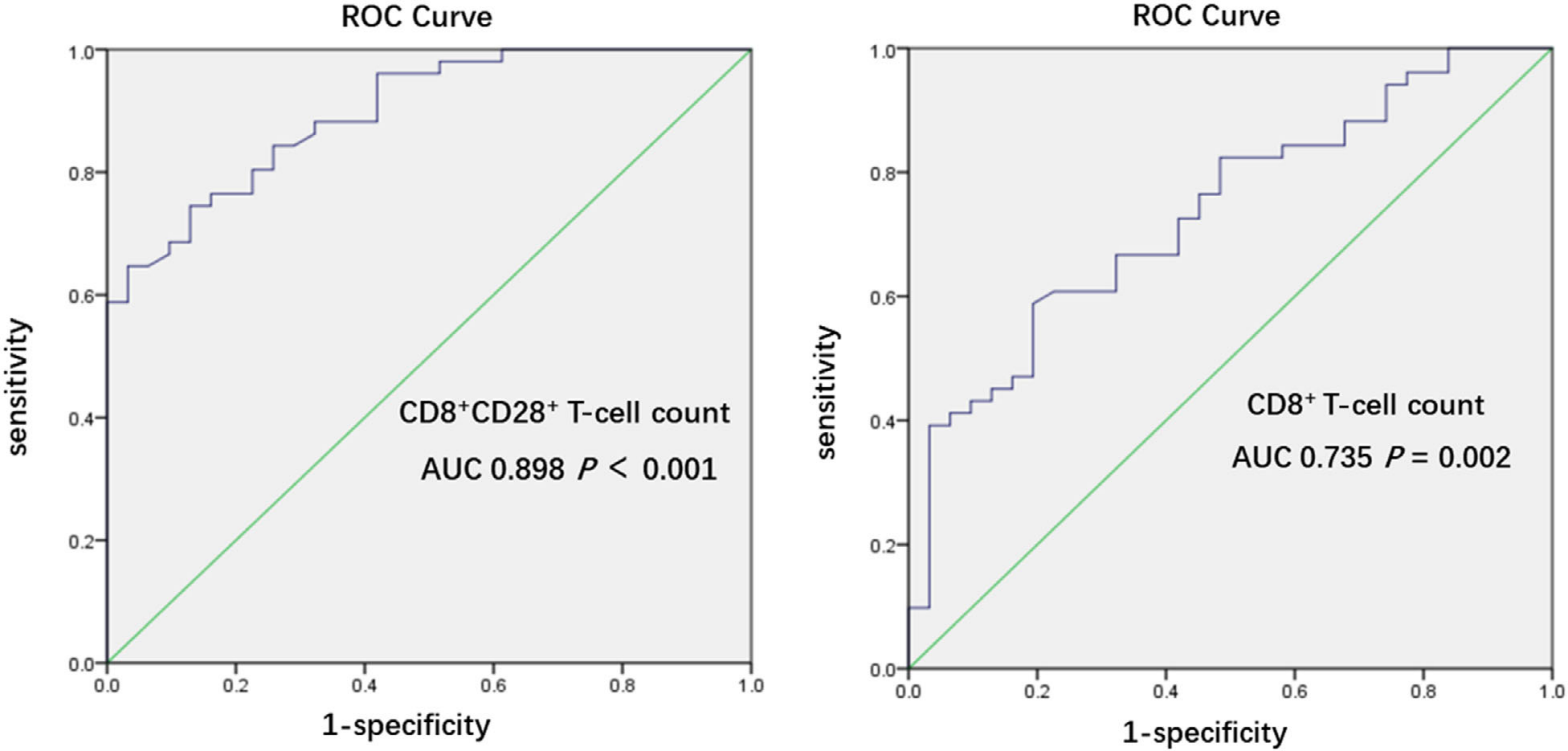
Receiver operating characteristic (ROC) curve analysis of parameters predicting cytomegalovirus‐DNA–negative conversion. The cutoff was obtained by calculating the optimal operating point, which is the value for the point on the curve that had the minimum distance to the upper left corner (where sensitivity = 1 and specificity = 1). In this ROC curve, the cutoff values of CD8^+^CD28^+^ and CD8^+^ T‐cell counts for predicting cytomegalovirus‐DNA–negative conversion were 151 cells mm^−3^ with a 95% confidence interval (CI) of 0.834–0.961 (*P* < 0.0001) and 358 cells mm^−3^ with a 95% CI of 0.627–0.844 (*P* < 0.0001), respectively. AUC, area under the curve.

To assess the effect of immune parameters on prognosis, all active CMV–infected patients with sepsis were further divided into CMV‐DNA–negative conversion and CMV‐DNA persistently positive groups. Among the immune parameters, only CD8^+^ and CD28^+^ T‐cell counts differed significantly between these two groups (Table 5). To avoid statistical error caused by multicollinearity, the CD8^+^ and CD8^+^CD28^+^ T‐cell counts were similarly performed in logistic regression, respectively. Regression analysis suggested that the CD8^+^CD28^+^ and CD8^+^ T‐cell counts were positively associated with CMV‐DNA–negative conversion (odds ratio 1.025 *versus* 1.005; *P* < 0.001 *versus P* = 0.002). The receiver operating characteristic curve analysis showed that compared with the CD8^+^ T‐cell count, the CD8^+^CD28^+^ T‐cell count had higher discriminatory power with an area under the curve of 0.898 (Figure 2 and Table 6). A CD8^+^CD28^+^ T‐cell count cutoff of 151 cells mm^−3^ at ICU admission may predict CMV‐DNA–negative conversion with a sensitivity of 74.5% and a specificity of 87.1%, which were obtained by calculating the Youden index (data are presented in Supplementary table 2).

**Table 5:** Immune parameters between CMV‐DNA persistently positive and negative conversion patients

Variables	CMV‐DNA+ (*n* = 82), median (IQR)	Negative conversion (*n* = 51), median (IQR)	Persistently positive (*n* = 31), median (IQR)	*P*‐value
WBC (10^9^ L^−1^)	10.45 (8.2)	9.90 (8.08)	10.87 (7.62)	0.257
NG (10^9^ L^−1^)	8.37 (7.44)	8.12 (7.91)	8.67 (7.56)	0.202
NK (cells mm^−3^)	60.5 (57)	59 (64)	61 (64)	0.852
B cell (cells mm^−3^)	101 (91.25)	93 (73)	113 (101)	0.579
Lymphocyte (cells mm^−3^)	891 (538.25)	990 (538)	819 (508)	0.115
CD4^+^ T	377 (352.75)	380 (333)	418 (382)	0.793
CD4^+^CD28^+^ T	351.5 (387.5)	377 (399)	301 (400)	0.488
CD4^+^CD28^−^ T	10 (54.25)	7 (29)	12 (109)	0.322
CD8^+^T	328 (224.75)	371 (230)	240 (194)	**< 0.0001**
CD8^+^CD28^+^ T	153.5 (171)	216 (141)	77 (87)	**< 0.0001**
CD8^+^CD28^−^ T	173.5 (111.75)	165 (110)	178 (112)	0.629
Complement factor (g L^−1^)					
C3	0.79 (0.40)	0.72 (0.37)	0.86 (0.34)	0.118
C4	0.17 (0.1)	0.15 (0.10)	0.19 (0.08)	0.297
Immunoglobulin (g L^−1^)					
IgA	2.29 (1.47)	2.15 (1.43)	2.39 (2.11)	0.688
IgG	10.6 (8.06)	11.42 (7.55)	10.6 (13.64)	0.789
IgM	0.76 (0.56)	0.75 (0.60)	0.73 (0.55)	0.322

CMV, cytomegalovirus; Ig, immunoglobulin; IQR, interquartile range; NG, neutrophil granulocyte; NK, natural killer (cells); WBC, white blood cell count.

Bold values are statistically significant.

**Table 6:** Multivariate logistic regression analysis of CD8^+^ T‐cell and CD8^+^CD28^+^ T‐cell count distinguishing CMV‐DNA–negative conversion, respectively

Parameters	OR	95% CI	*P*‐value
CD8^+^ T‐cell count	1.005	1.002–1.009	**0.002**
CD8^+^CD28^+^ T‐cell count	1.025	1.014–1.036	**< 0.0001**

CI, confidence interval; CMV, cytomegalovirus; OR, odds ratio.

Bold values are statistically significant.

### Comparison of parameters in surviving and nonsurviving active CMV–infected patients with sepsis

**Figure 3:**
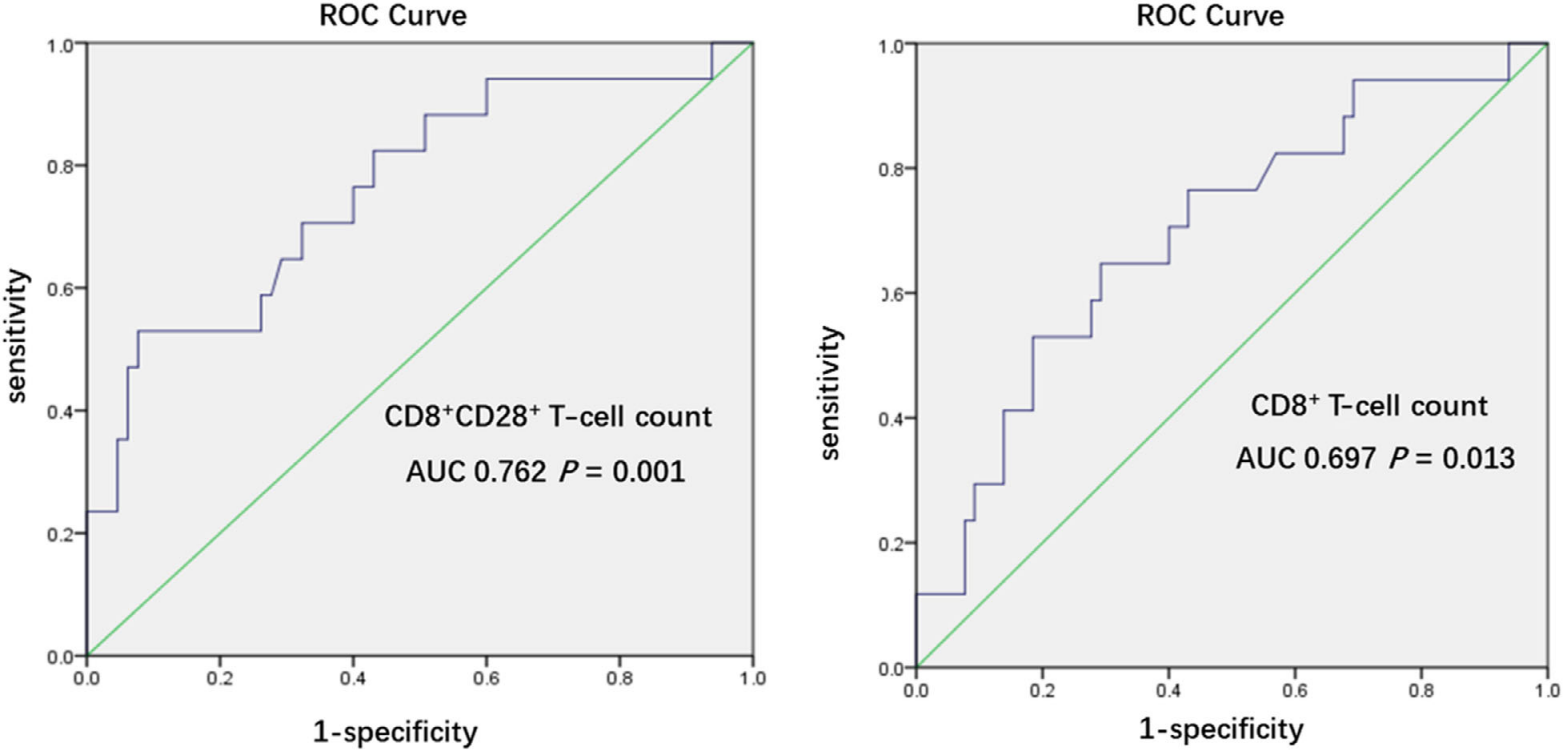
Receiver operating characteristic (ROC) curve analysis of parameters predicting 28‐day mortality. Similarly, in this ROC curve, the cutoff values of the CD8^+^CD28^+^ and CD8^+^ T‐cell counts for predicting 28‐day mortality were 64.5 cells mm^−3^ with a 95% confidence interval (CI) of 0.627–0.897 (*P* < 0.01) and 266 cells mm^−3^ with a 95% CI of 0.555–0.839 (*P* < 0.05). AUC, area under the curve.

**Figure 4:**
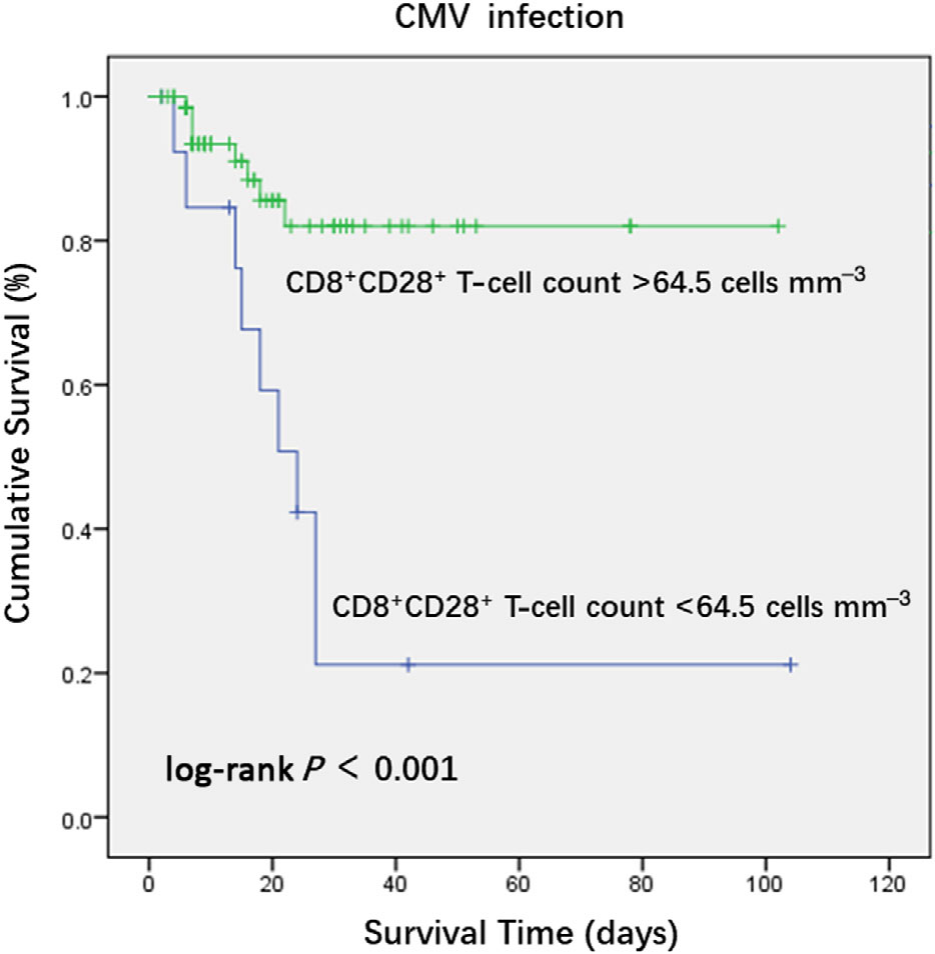
Kaplan–Meier analysis of survival probabilities of patients with cytomegalovirus (CMV) infection. Survival was measured according to the CD8^+^CD28^+^ T‐cell count < 64.5 cells mm^−3^ at intensive care unit admission. Survival time censored on day 28.

We divided all active CMV–infected patients with sepsis into survivor and nonsurvivor groups based on their 28‐day mortality after enrollment. The CD8^+^ and CD28^+^ T‐cell counts showed significant differences, whereas other immune parameters showed no significant differences between the two groups (Table 7). According to logistic regression analysis, the CD8^+^CD28^+^ and CD8^+^ T‐cell counts were negatively correlated with 28‐day mortality after enrollment (Table 8). Similarly, receiver operating characteristic curve analysis was conducted to compare the predictive efficacy of the two parameters. The CD8^+^CD28^+^ T‐cell count may have been more accurate in predicting the 28‐day mortality after enrollment for active CMV–infected patients with sepsis. A lower CD8^+^CD28^+^ T‐cell count may have been associated with a higher 28‐day mortality. The sensitivity and specificity of a cutoff value of 64.5 cells mm^−3^ in predicting the 28‐day mortality of active CMV–infected patients with sepsis were 52.9% and 92.3%, respectively (Figure 3). The Kaplan–Meier analysis showed that active CMV–infected patients with sepsis with CD8^+^CD28^+^ T‐cell count < 64.5 cells mm^−3^ (log‐rank *P* < 0.001) were associated with a lower survival rate in the ICU (Figure 4).

**Table 7:** Immune parameters in all patients with CMV infection according to 28‐day mortality

Variables	CMV (*n* = 82)	Survivors (*n* = 59)	Nonsurvivors (*n* = 23)	*P‐*value
WBC (10^9^ L^−1^)	10.45 (8.2)	10.31 (8.03)	10.59 (11.47)	0.194
NG (10^9^ L^−1^)	8.37 (7.44)	8.32 (6.99)	8.41 (10.49)	0.255
NK (cells mm^−3^)	60.5 (57)	59 (60)	61 (49)	0.895
B cell (cells mm^−3^)	101 (91.25)	97 (85)	95 (149)	0.864
Lymphocyte (cells mm^−3^)	891 (538.25)	945 (532)	774 (377)	0.085
CD4^+^T	377 (352.75)	380 (401)	418 (288)	0.503
CD4^+^CD28^+^ T	351.5 (387.5)	365 (385)	325 (400)	0.158
CD4^+^CD28^−^ T	10 (54.25)	8 (38)	57 (170)	0.169
CD8^+^ T	328 (224.75)	358 (221)	228 (168)	**0.013**
CD8^+^CD28^+^ T	153.5 (171)	189 (170)	64 (102)	**< 0.0001**
CD8^+^CD28^−^ T	173.5 (111.75)	177 (103)	142 (70)	0.417
Complement factor (g L^−1^)				
C3	0.79 (0.40)	0.80 (0.45)	0.71 (0.20)	0.615
C4	0.17 (0.1)	0.18 (0.11)	0.16 (0.07)	0.415
Immunoglobulin (g L^−1^)				
IgA	2.29 (1.47)	2.25 (1.58)	1.76 (2.8)	0.351
IgG	10.6 (8.06)	11.42 (8.13)	10.6 (13.38)	0.877
IgM	0.76 (0.56)	0.74 (0.56)	0.73 (0.67)	0.918

Data are presented as *n* (%) or median (IQR).

CMV, cytomegalovirus; Ig, immunoglobulin; IQR, interquartile range; NG, neutrophil granulocyte; NK, natural killer (cells); WBC, white blood cell count.

Bold values are statistically significant.

**Table 8:** Multivariate logistic regression analysis of CD8^+^CD28^+^ T‐cell and CD8^+^ T‐cell count distinguishing 28‐day mortality, respectively

Parameters	OR	95% CI	*P*‐value
CD8^+^CD28^+^ T‐cell count	0.990	0.983–0.997	**0.007**
CD8^+^ T‐cell count	0.996	0.992–1.000	**0.036**

CI, confidence interval; OR, odds ratio.

Bold values are statistically significant.

## DISCUSSION

To our knowledge, this is the first prospective study to explore the relationship between host immune phenotype and prognosis (including CMV‐DNA–negative conversion and 28‐day mortality) in patients with sepsis with active CMV infection. In addition, as CMV can remain latent in the body for long periods and because it is hard to get clear evidence of past CMV infection, we divided our patients into active CMV–infected and nonactive CMV–infected groups according to the clinicians’ practice logic. Compared with patients without active CMV infection, ICU, in‐hospital and 28‐day mortalities were significantly increased in active CMV–infected patients with sepsis. Both the CD8^+^CD28^+^ and CD8^+^ T‐cell counts could be combined with the NK cell count, Acute Physiology and Chronic Health Evaluation II score and immunoglobulin G as related factors for active CMV infection in patients with sepsis. The CD8^+^CD28^+^ T‐cell count was the best predictor of CMV‐DNA–negative conversion potential in active CMV–infected patients with sepsis. A CD8^+^CD28^+^ T‐cell count cutoff of 151 cells mm^−3^ at ICU admission may predict the CMV‐DNA–negative conversion with a sensitivity of 74.5% and a specificity of 87.1%. A lower CD8^+^CD28^+^ T‐cell count may be associated with a higher 28‐day mortality. The sensitivity and specificity of a CD8^+^CD28^+^ T‐cell count cutoff value of 64.5 cells mm^−3^ in predicting the 28‐day mortality of active CMV–infected patients with sepsis were 52.9% and 92.3%, respectively. Previous studies^12^, ^13^ have demonstrated that the host innate immune system, specific NK cells, multiple antibodies and effector/memory T‐cell responses are activated rapidly by viruses which block the continuous replication of CMV DNA, and that CD8^+^ T cells are recruited to the infected site to eliminate the virus by releasing cytokines. These conclusions are consistent with our research, as we found that the NK cell count, immunoglobulin G and the CD8^+^CD28^+^ T‐cell count in active CMV–infected patients with sepsis were significantly higher than in those without active CMV infection. In addition, a recent study by Pickering *et al*.^14^ found that NK cells and CD8^+^ T cells play an important role in the process of CMV infection in patients after renal transplantation and NK cells are closely related to the development of CMV infection while CD8^+^ T cells are essential for the control of CMV. Their results were consistent with our findings. We think that the low count of NK cells may reflect the disorder of host immune status, which is in line with the immune characteristics of patients with sepsis and may explain why they are more likely to be infected by CMV and have a poor prognosis. However, we did not find a significant difference in the CD4^+^ T‐cell count between active CMV–infected and nonactive CMV–infected patients with sepsis, which is different from that found by Kaplan *et al*.^5^ The reason for this difference may be related to the different patients in the two studies. CD4^+^ T cells are the only specific target of HIV, and the CD4^+^ T‐cell count of HIV‐infected patients is associated with susceptibility to opportunistic pathogens. Therefore, the CD4^+^ T‐cell count could specifically represent the immune status of HIV‐infected patients.^15^ Our study involved patients with sepsis, in whom immune status was affected by various factors such as underlying diseases and treatment. Moreover, a study analyzing cellular immunity after CMV infection by Radha *et al*.^16^ showed that, although CMV infection was mainly controlled by CD8^+^ and CD4^+^ T cells, CD8^+^ T‐cell response seemed to play a more important role in this process. By comparing T‐cell proliferation after CMV infection, they found that under the same antiviral treatment, patients with rapid proliferation of CD8^+^ T cells had faster CMV‐DNA–negative conversion.

The anti‐CMV activity of CD8^+^ T cells is closely related to their high sensitivity to CMV peptide fragments. It has been reported that approximately 30% of CD8^+^ T cells in healthy adults are CMV reactive.^17^ After the invasion of CMV, CD8^+^ naïve T cells are rapidly amplified and differentiate into different effector cells upon recognition of CMV‐specific polypeptides such as CMV tegument protein 65 (pp65) and immediate‐early protein 1,^18^ costimulators such as CD28 and CD137,^19^ and cytokines such as interleukin‐2 and interleukin‐15.^20^, ^21^ These effector cells then circulate into the tissues and perform antiviral functions through perforin‐ and interferon‐γ–mediated mechanisms.^22^ As an important costimulator of T cells, CD28 is involved in the activation and differentiation of CD8^+^ T cells and is a typical surface marker that combines with others to classify functional subsets.^23^, ^24^ The costimulative effect of CD28 accelerates the response speed of T cells and is essential for host resistance to life‐threatening acute viral diseases.^25^, ^26^ A study by Zielinski and colleagues has demonstrated that CD8^+^CD28^+^ T cells have good predictive value for the incidence of CMV infection in kidney transplantation patients.^27^ Patients with a lower CD8^+^CD28^+^ T‐cell count have a higher incidence of CMV infection despite receiving prophylaxis after kidney transplantation surgery. In addition, it has been demonstrated that expression of CD28 on the surface of T cells is significantly decreased in patients with severe infection, and the reduction of CD28 expression may be an important risk factor for high mortality.^28^ Our findings are consistent with the aforesaid conclusions. First, by comparing the immune parameters of all patients with sepsis, we found that CD8^+^CD28^+^ T cells may be an important related factor for active CMV infection in patients with sepsis. We also found that, among all active CMV–infected patients with sepsis, those with a lower CD8^+^CD28^+^ T‐cell count had less CMV‐DNA–negative conversion and a higher 28‐day mortality after enrollment. Lastly, logistic regression and receiver operating characteristic curve analysis showed that the CD8^+^CD28^+^ T‐cell count was a better predictor of prognosis than the CD8^+^ T‐cell count for active CMV–infected patients with sepsis. These results indicate that CD8^+^ CD28^+^ T cells may play an irreplaceable and important role in host resistance to CMV, providing new evidence for better evaluation of prognosis in active CMV–infected patients with sepsis.

CD8^+^CD28^−^ T cells also play an important role in host resistance to CMV infection.^29^ Studies have demonstrated that CD8^+^CD28^−^ T cells continue to expand when hosts are chronically exposed to CMV, providing lasting defense as effector memory cells^30^ and suppressing the excessive T regulatory cell response to CMV,^31^ to maintain immune balance. This is the main reason why CMV could be latent for the lifetime of humanity.^32^ Therefore, CD8^+^CD28^−^ T cells are currently considered to play a major role in the long‐term defense of host resistance to CMV infection.^13^ Although CMV‐infected patients were all at the CMV‐DNA replication stage, we did not further distinguish the host immune phase in resistance to CMV infection. This may be one of the reasons for there being no difference in the CD8^+^CD28^−^ T‐cell count between the groups. Another explanation for the lack of difference in the CD8^+^CD28^−^ T‐cell count between the groups in our study is age. Numerous studies^33^, ^34^, ^35^ have indicated that CD8^+^CD28^−^ T cells are associated with immune senescence, and the proportion of CD8^+^ T cells lacking CD28 surface expression increases with age, which may explain the increased susceptibility of elderly individuals. However, in our study, there was no significant difference in age between the groups.

Our study had several limitations. First, the specific mechanism between CMV replication and poor prognosis in patients with sepsis remains unclear. An animal study by Cook *et al*.^36^ speculated that reactivation of CMV may induce significant activation of proinflammatory factors such as tumor necrosis factor‐α during sepsis. In addition, Barton *et al*.^37^ found that CMV infection may activate the mononuclear macrophage system in mice, which plays an important role in immune defense. These studies suggest that CMV reactivation may have the potential to serve as a sepsis‐related proinflammatory factor and immune activation indicator, and demonstrate that bone marrow–derived cells are activated by CMV infection and differentiated into specific functional effector cells. A recent study by Hassouneh *et al*.^38^ indicated that CMV infection leads to functional changes in T‐cell subtypes. Therefore, it is reasonable to assume that CMV infection may impair cytotoxic lymphocyte response to CMV, which in turn contributes to poor prognosis in patients with sepsis. Second, CMV‐specific CD8^+^ and CD4^+^ T cells were not selected for subpopulation analysis because the purpose of this study was to explore the role of host immune phenotype in CMV‐infected patients with sepsis. Thus, we selected the most common and easily available immune indicators for analysis. Third, as both initial CMV infection and reactivation are included in CMV infection, we did not distinguish the onset type of CMV infection. Lastly, previous studies have shown that some human CMV (HCMV) subtypes that are genetically distinct and phenotypically unique may have some effect on the course of disease, or even be associated with clinical outcome in specific patient groups.^39^, ^40^ However, we did not evaluate this because of limited laboratory conditions. In addition, we did not select multiple time nodes to longitudinally analyze the possible correlation between host immune phenotype at different stages of sepsis and CMV infection. In the future, we plan to group patients according to CMV initial infection/reactivation, analyze host immune phenotype of CMV‐specific T cells, try to identify CMV subtypes and collect data from multiple time nodes to explore the relationship between CMV infection and host immune phenotype of patients with sepsis.

In conclusion, we found that the CD8^+^CD28^+^ T‐cell count is associated with early CMV infection in patients with sepsis and has predictive potential for CMV‐DNA–negative conversion and 28‐day mortality. A higher CD8^+^CD28^+^ T‐cell count is associated with incidence of active CMV infection and lower 28‐day mortality in patients with sepsis. Our findings highlight the important role of host immune phenotype in active CMV–infected patients with sepsis and provide strong evidence that T‐lymphocyte subtyping could facilitate early diagnosis and prognosis of active CMV infection and help identify patients with sepsis having a high mortality risk.

## METHODS

### Study population and design

Patients with sepsis hospitalized in the ICU at Peking Union Medical College Hospital between February 2017 and July 2020 were assessed in this prospective study (Figure 1). Informed consent was obtained from all the patients involved. This study was approved by the Ethics Committee of Peking Union Medical College Hospital and registered in the Chinese Clinical Trial Register (ChiCTR; identifier ChiCTR‐ROC‐17010750).

Inclusion criteria were as follows: (1) age > 18 years; (2) ICU stay ≥ 48 h and (3) diagnosis of sepsis. Sepsis was defined as life‐threatening organ dysfunction resulting from host immune imbalance caused by infection. Infection was diagnosed by an intensivist and met the systemic inflammatory response syndrome criteria. Organ dysfunction was defined as Sequential Organ Failure Assessment score ≥ 2 as a result of the infection.^1^ The exclusion criteria were (1) immunosuppression caused by organ or hematopoietic stem cell transplantation; (2) HIV infection; (3) immunosuppressive treatment prior to admission, that is, prednisone 0.5 mg kg^−1^ day^−1^ at least 2 weeks before the study, and tumor necrosis factor antagonist, methotrexate or chemotherapy for cancer within 4 weeks of the study; (4) treatment with antiviral drugs active against CMV, such as ganciclovir or valganciclovir before admission and (5) pregnancy or lactation.

Patients enrolled in this study underwent a thorough physical examination and all necessary blood and other specimens for laboratory work were collected while the patients were admitted in the ICU to determine the site of infection and the causative pathogen, and to eliminate bacterial colonization. The infection foci were confirmed according to the following conditions. Pneumonia^41^ was diagnosed clinically as new or progressive pulmonary infiltrates caused by infection with at least two manifestations as follows: fever > 38°C or hypothermia < 36°C; leukocytosis (> 12 000 cells mm^−3^) or leukopenia (< 4000 cells mm^−3^) and presence of newly purulent tracheal secretions and hypoxia. Bloodstream infection was defined as symptoms of infection combined with the presence of a typical or atypical pathogen in blood culture.^42^ Abdominal infection was diagnosed as a new or progressive manifestation of peritonitis, such as abdominal tenderness or rebound pain, accompanied by increased ascites and/or changes in its nature.^43^ Skin and soft‐tissue infection was diagnosed as soft‐tissue infection accompanied by signs and symptoms of systemic toxicity.^44^ Other infections were mediastinal infection and biliary or urinary tract infection, which have been described previously.^45^

Blood was sampled simultaneously from at least two different parts. For collecting the sputum samples, patients were tracheal intubated. The aspirated samples met the standard for lower‐tract samples of > 25 white blood cells and < 10 epithelial cells per low‐power field of view. The tissue specimen was sampled by the surgeon for culture and then the incisions were fully sterilized. Urine samples were acquired midstream and obtained after placing or changing the catheter.

Active CMV infection was defined as first detection of serum CMV DNA ≥ 500 copies mL^−1^ by real‐time quantitative PCR, with or without clinical manifestations.^5^, ^46^ As CMV load is closely related to the severity of infection and the efficacy of treatment,^47^ in this research patients with sepsis were first divided into active CMV–infected and nonactive CMV–infected groups. Active CMV–infected patients with sepsis were further divided into CMV‐DNA–negative conversion and CMV‐DNA persistently positive groups. Serum CMV‐DNA retesting was performed per week in patients with active CMV infection after they received a therapeutic approach during ICU/inpatient treatment. CMV‐negative conversion was defined as retesting serum CMV DNA > 500 copies mL^−1^ during hospitalization. Persistently positive CMV was defined as > 500 copies mL^−1^ of retesting serum CMV DNA.

Follow‐up included length of ICU and in‐hospital stay as well as ICU, in‐hospital and 28‐day mortality after enrollment. There was no standard treatment for critical illness, and all treatment options and drug choices were determined by the clinicians.

### Clinical and laboratory evaluation

#### Clinical assessments

All patients underwent a comprehensive clinical evaluation on the day of admission to the ICU. Age, sex, underlying disease and important infectious and biochemical indicators (procalcitonin, creatinine, albumin and bilirubin) were all included. All the specimens were immediately sent to the Clinical Laboratory of Peking Union Medical College Hospital, an internationally recognized advanced laboratory certified by ISO15189 and the College of American Pathologists for examination. CMV‐DNA levels were determined as well as Acute Physiology and Chronic Health Evaluation II^48^ and Sequential Organ Failure Assessment scores.^49^ Life‐sustaining treatments (need for mechanical ventilation, vasopressors or renal replacement therapy) for ≥ 24 h were recorded based on clinical evaluation and recent recommendations.^50^ The Third International Consensus Guidelines on the Management of Cytomegalovirus in Solid‐Organ Transplantation^51^ in 2018 suggested that once CMV infection occurs in patients with sepsis, it progresses rapidly, and treatment for CMV should be initiated as soon as possible. Thus, intravenous infusion of ganciclovir 5 mg kg^−1^ Q12h and observe the change of serum CMV‐DNA levels during treatment as the guideline recommended is the therapeutic approach applied to the patients in this study.^51^

#### Cytomegalovirus detection

The CMV‐DNA Diagnostic Kit was used to detect the CMV‐DNA level in plasma (per 100 μL) by real‐time quantitative PCR using the Therma‐Base Taqman technology in the NATCH S automatic nucleic acid extraction system. To amplify HCMV DNA, the following primer and fluorogenic probe sequences were located in the *UL83* gene: pp549s (direct primer), 5′‐GTCAGCGTTCGTGTTTCCCA‐3′; pp812as (reverse primer), 5′‐GGGACACAACACCGTAAAGC‐3′ and pp770s (fluorogenic probe), 5′ FAM‐CCCGCAACCCGCAACCCTTCATG‐3′ TAMRA.^52^ HCMV‐negative and HCMV‐positive control samples were used as contrast agents for qualitative detection, whereas plasmid pKS‐pp65K7 was used to construct the standard curve for HCMV DNA quantification.^53^ In brief, 10‐fold dilutions of the plasmid standard pKS‐pp65K7 containing a cloned insert of the target sequence were prepared ranging from 4.00 × 10^2^ to 4.00 × 10^9^ copies mL^−1^, which is the detection threshold of the CMV‐DNA PCR Kit. Then, according to ISO 15189^54^ and College of American Pathologists documents, the CMV‐DNA copy number > 500 copies mL^−1^ was defined as CMV‐DNA positive through the performance verification of reagents. The kit, system, control samples and HCMV‐DNA fragments were all purchased from Shengxiang Biotechnology Co., Ltd (Hunan, China).

#### Immunological laboratory examination

**Figure 5:**
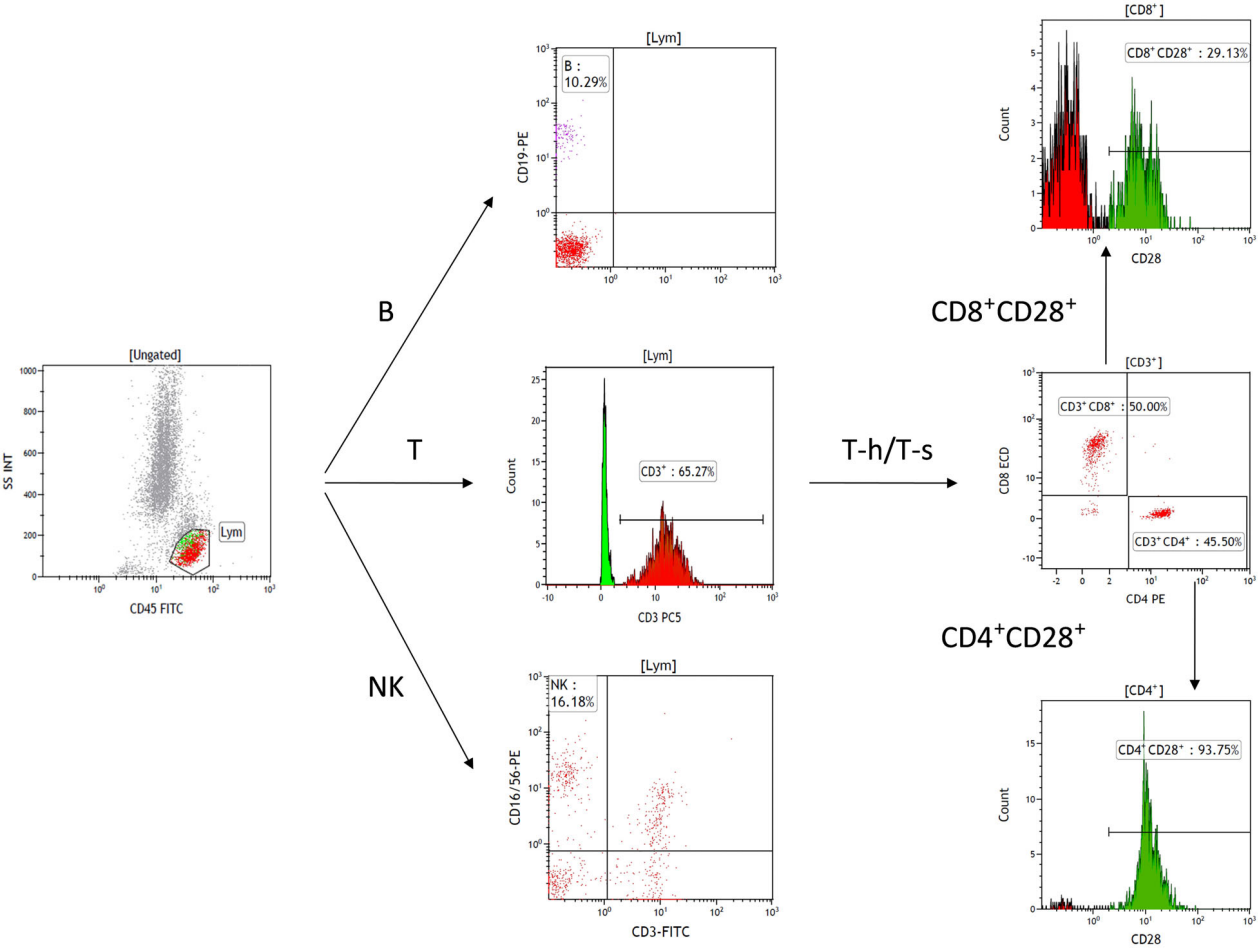
The gating strategy of flow cytometry experiments. FITC, fluorescein isothiocyanate; FS, forward scatter; INT, integral; Lym, lymphocytes; NK, natural killer (cells); PE, phycoerythrin; SS, side scatter.

**Figure 6:**
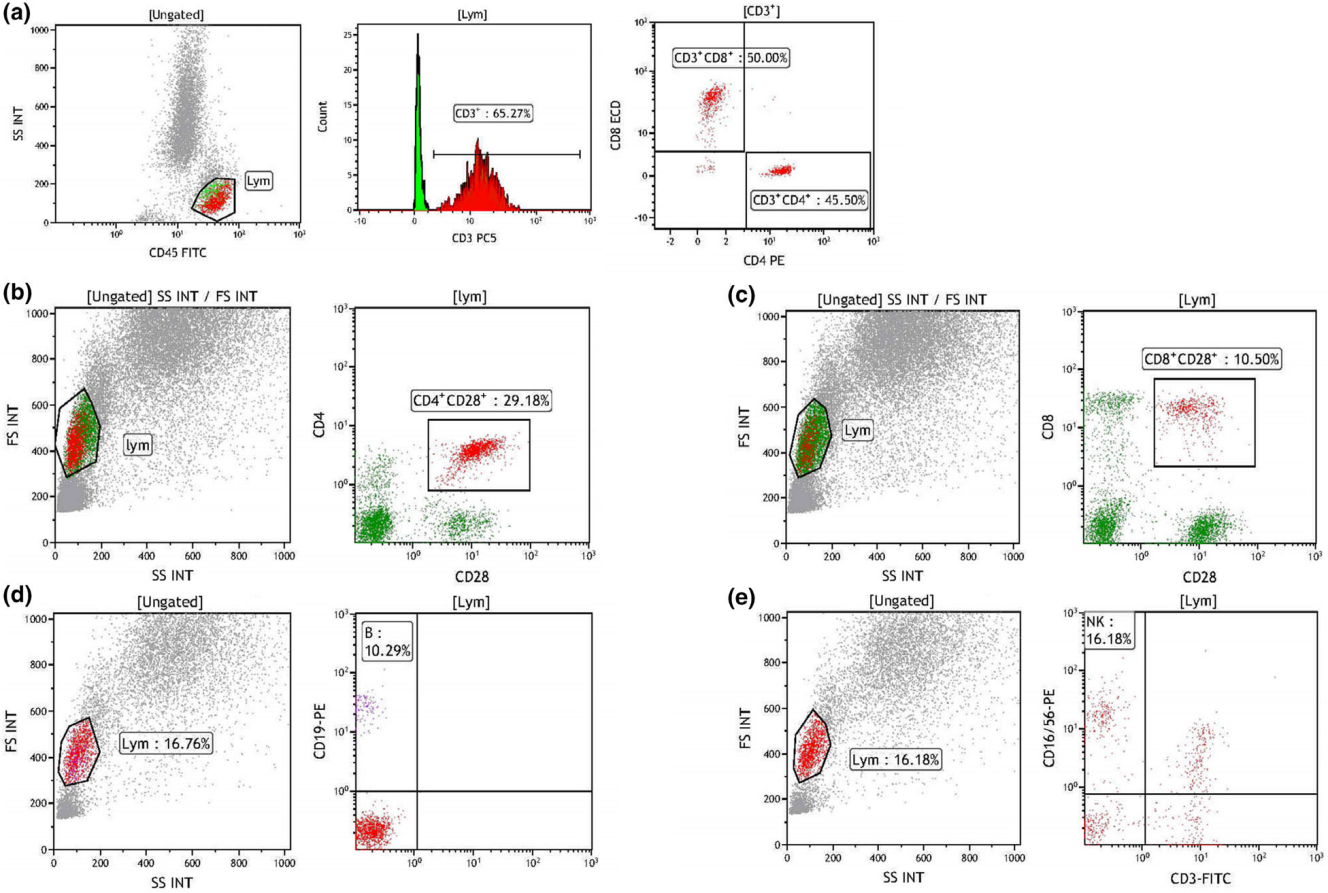
Sample flow cytometry experiment data. **(a)** Sample gating data on T lymphocytes. The gating percentages of [CD3^+^], [CD3^+^CD4^+^] and [CD3^+^CD8^+^] were 65.27%, 45.5% and 50.0%, respectively. **(b)** The gating percentage of [CD4^+^CD28^+^] was 29.18%. **(c)** The gating percentage of [CD8^+^CD28^+^] was 10.5%. **(d)** The gating percentage of B cell was 10.29%. **(e)** The gating percentage of NK cell was 16.18%. FITC, fluorescein isothiocyanate; FS, forward scatter; INT, integral; Lym, lymphocytes; NK, natural killer (cells); PE, phycoerythrin; SS, side scatter.

As previously described,^55^ for flow cytometry, peripheral blood mononuclear cells were isolated and stained with combinations of different fluorescent monoclonal antibodies, followed by flow cytometric analysis to detect T cells (CD3^+^), CD4^+^ T‐cell subsets (CD4^+^CD3^+^ and CD4^+^CD28^+^), CD8^+^ T‐cell subsets (CD8^+^CD3^+^, CD8^+^CD28^+^), B cells (CD19^+^) and NK cells (CD3^−^CD16^+^ CD56^+^). The gating strategy of flow cytometry experiments is shown in Figures 5 and 6. Details of the equipment and reagents used in flow cytometric analysis are as follows: flow cytometer, Beckman Coulter NAVIOS; analysis software, Kaluza; antibodies, CD45‐FITC/CD3‐PC5/CD4‐RD1/CD8‐ECD (Beckman Coulter, Brea, CA, USA, 6 607 013); CD3‐FITC/CD (16 + 56)‐PE (Beckman Coulter, Brea, CA, USA, A07735); CD19‐PE (Beckman Coulter, Brea, CA, USA, A07769); CD4‐FITC (Beckman Coulter, Brea, CA, USA, A07750); CD8‐FITC (Beckman Coulter, Brea, CA, USA, A07756); CD28‐PE (Beckman Coulter, Brea, CA, USA, IM2071U); side light scatter *versus* CD45 was used to gate lymphocytes. The lymphocytes were further divided into B cell (CD19^+^), T‐helper cell (CD3^+^CD4^+^), cytotoxic T lymphocyte cell (CD3^+^CD8^+^), CD4^+^CD28^+^ T‐cell (CD4^+^CD28^+^), CD8^+^CD28^+^ T‐cell (CD8^+^CD28^+^) and NK cell (CD3^−^CD16/56^+^). Peripheral blood was extracted and serum obtained by centrifugation for the detection of complement and immunoglobulin. An AU5800 automatic biochemical analyzer (Beckman Coulter, Brea, CA, USA) was used to detect the change of absorbance by immunity transmission turbidity.

### Statistical analysis

All analyses were performed using SPSS for Windows version 24.0 (IBM Corp., Armonk, NY, USA). The Kolmogorov–Smirnov test, Student's *t* test and the Mann–Whitney *U*‐test were used to analyze the variables. The Chi‐squared or Fisher's exact test was chosen for comparison of categorical variables. Variables that showed *P* < 0.05 in univariate analysis were included in the multivariate regression analysis model. Principal component analysis was applied to adjust for the possible multicollinearity among variables and the results were expressed as Wald index, *P*‐value and odds ratio with 95% confidence intervals. The discriminatory ability of immune parameters for predicting 28‐day mortality was determined by receiver operating characteristic curve analysis. The reliabilities and consistencies of diagnostic tests were assessed by calculating their sensitivity, specificity and positive and negative predictive values. The Kaplan–Meier survival analysis was used to construct survival curves, and comparisons of survival distributions were based on the log‐rank test. All tests performed were two‐tailed, with *P* < 0.05 considered to be statistically significant.

### ACKNOWLEDGMENTS

We are grateful to all the patients with sepsis who participated in this study. We also acknowledge the staff at the ICU of Peking Union Medical College Hospital. We thank Cathel Kerr from Liwen Bianji, Edanz Editing China (www.liwenbianji.cn/ac) for editing the English text of a draft of this manuscript. The work was supported by the Beijing Municipal Science and Technology Commission (No. Z201100005520049), the National Natural Science Foundation of China (No. 82072226), the National Key R&D Program of China 2022YFC2009803 from the Ministry of Science and Technology of the People's Republic of China, the CAMS Innovation Fund for Medical Sciences (CIFMS) 2021‐I2M‐1‐062 from the Chinese Academy of Medical Sciences.

## CONFLICT OF INTEREST

All authors declare that they have no conflicts of interest.

## ETHICS APPROVAL AND CONSENT TO PARTICIPATE

This study was approved by the local institutional review board of Peking Union Medical College Hospital (Ethics approval number: JS‐1170). Informed consent was obtained from all the patients involved.

## AUTHOR CONTRIBUTIONS

**Guangxu Bai:** Conceptualization; data curation; formal analysis; software; visualization; writing – original draft; writing – review and editing. **Na Cui:** Conceptualization; data curation; formal analysis; funding acquisition; methodology; project administration; supervision; writing – review and editing. **Hao Wang:** Conceptualization; data curation; project administration; supervision; writing – review and editing. **Wei Cheng:** Data curation; formal analysis; methodology; writing – original draft. **Wen Han:** Data curation; formal analysis; methodology; writing – original draft. **Jianwei Chen:** Data curation; formal analysis; methodology; writing – original draft. **Ye Guo:** Formal analysis; methodology. **Fei Wang:** Formal analysis; methodology.

## Data Availability

The data that support the findings of this study are available on request from the corresponding author. The data are not publicly available due to privacy or ethical restrictions.
