# Supplementary material for: T‐lymphocyte subtyping: an early warning and a potential prognostic indicator of active cytomegalovirus infection in patients with sepsis
Source: Immunol Cell Biol. 2022 Oct 12;100(10):777–90. doi: 10.1111/imcb.12586 (PMC9828035; doi:10.1111/imcb.12586)
Supplement: Supplementary file 1 [file IMCB-100-777-s001.docx]

**Supplementary table 1. Multivariate logistic regression analysis of factors distinguishing CMV infection**

| Parameters | OR | 95%CI | *P-*value |
| --- | --- | --- | --- |
| Fungal infection | 0.563 | 0.294-1.076 | 0.082 |
| CPIS | 1.090 | 0.991-1.199 | 0.074 |
| APACHE II | 1.048 | 1.011-1.086 | **0.010** |
| NK | 0.992 | 0.988-0.997 | **0.001** |
| IgG | 1.073 | 1.025-1.123 | **0.002** |
| CD8^+^ T cell counts | 1.001 | 1.000-1.002 | **0.008** |

*APACHE II* Acute Physiology And Chronic Health Evaluation II; *CI* confidence interval; *NK* natural killer cell; *OR* odds ratio.

**Supplementary table 2.** **CD8^+^CD28^+^ T cell count cutoff of 151 cells/mm3 at ICU admission may predict CMV-DNA negative conversion with a sensitivity of 74.5% and specificity 87.1% which were obtained by calculating the Youden index.**
